# baySeq: Empirical Bayesian methods for identifying differential expression in sequence count data

**DOI:** 10.1186/1471-2105-11-422

**Published:** 2010-08-10

**Authors:** Thomas J Hardcastle, Krystyna A Kelly

**Affiliations:** 1 Department of Plant Sciences, University of Cambridge, Downing Street, Cambridge, UK

## Abstract

**Background:**

High throughput sequencing has become an important technology for studying expression levels in many types of genomic, and particularly transcriptomic, data. One key way of analysing such data is to look for elements of the data which display particular patterns of differential expression in order to take these forward for further analysis and validation.

**Results:**

We propose a framework for defining patterns of differential expression and develop a novel algorithm, baySeq, which uses an empirical Bayes approach to detect these patterns of differential expression within a set of sequencing samples. The method assumes a negative binomial distribution for the data and derives an empirically determined prior distribution from the entire dataset. We examine the performance of the method on real and simulated data.

**Conclusions:**

Our method performs at least as well, and often better, than existing methods for analyses of pairwise differential expression in both real and simulated data. When we compare methods for the analysis of data from experimental designs involving multiple sample groups, our method again shows substantial gains in performance. We believe that this approach thus represents an important step forward for the analysis of count data from sequencing experiments.

## Background

The development of high-throughput sequencing technologies in recent years [1-4] has led to a massive increase in genomic data represented by *counts*. These count data are distinct from those acquired using bead and array technologies in that they are fundamentally discrete, rather than continuous, in nature. Rather than measurements of intensity, we acquire counts of the number of times a particular sequence is observed in a library, whether the source is genomic DNA, DNA fragments produced by immunoprecipitation, mRNA or small RNAs. Analyses of such sequence data are often concerned with detecting differential representation, that is, the discovery of data which are differentially represented between sets of biological replicates, particularly, but not exclusively, in analyses of transcriptomic data. These analyses are often challenging due to the small sample sizes available as a consequence of the relatively high cost of sequencing experiments.

This type of data first emerged from the serial analysis of gene expression (SAGE) [5], and a number of approaches were put forward for its analysis. Most of the early methods did not properly allow for replication or, when they did, could only be used to compare two groups. Baggerly *et al *[6] and Lu *et al *[7] introduced modelling approaches based on the overdispersed logistic and overdispersed log-linear distributions respectively that are able to handle both replicate data and multiple comparisons between groups. Robinson and Smyth derived an 'exact test' method based on the negative binomial distribution [8], and further developed this approach using a moderated test statistic sharing information across genomic locations to stabilize dispersion estimation in small samples [9]. This approach showed improvements in accuracy compared with the overdispersed logistic and log-linear approaches, but the methods are limited to pairwise comparisons. A recently developed method, DEGseq[10] takes an alternative approach, assuming normality of the log-ratios of the data from different biological samples conditional on the log geometric mean of the data. Another recent method DESeq[11] also makes the assumption of a negative binomial distribution, but adds the assumption of a locally linear relationship between over-dispersion and mean expression levels of the data. These later methods have not yet been fully described, but again appear strictly limited to pairwise comparisons.

We develop here an empirical Bayesian approach that is able to increase the accuracy of predictions by borrowing information across the dataset, but which removes the restriction of only considering pairwise comparisons and allows us to analyse more complex experimental designs. We are able to show that our method gives equivalent or improved performance in both simulated and biological data when compared to existing methods for the discovery of differential expression in pairwise comparisons, and offers improvements in performance for more complex designs.

In order to address the problem of more complex experimental designs involving multiple groups of samples, we develop our method in a very general form by first establishing a framework for describing diverse patterns of differential expression within a dataset. Using this framework to define a set of models, we seek to establish posterior probabilities of each model. Finally, we demonstrate the applicability of our method to these experimental designs on simulated data, and are able to show substantial improvements in performance using our method.

## Methods

We adopt and adapt the nomenclature of Robinson and Smyth [9] to describe SAGE data as this seems generally applicable to the data from high-throughput sequencing technologies. A set of data acquired by sequencing a cDNA *library *contains a number of sequence tags. Since in SAGE data, there is only one tag per mRNA molecule, Robinson and Smyth [9] examine methods for detecting differentially expressed tags between samples. However, in a number of applications made possible by high-throughput sequencing, we may wish to group multiple tags together and acquire a single count for that grouping. For example, with whole transcriptome mRNA or small RNA data, we may wish to consider the total number of counts for all tags coming from a defined locus. In either case, for each distinct tag or grouping of tags, we have an ordered list, or *tuple*, of discrete counts with the sample order the same in each tuple. In the work that follows, we therefore refer simply to *tuples*, without needing to specify whether these are counts of individually sequenced tags or aggregated counts of multiple tags. The *library size *is a measure of the total number of counts in a given library, or some surrogate measure of library size as discussed by Bullard *et al *[12], and is used as a scaling factor for the observed data.

### Approach

We take an empirical Bayesian approach to estimate the posterior probabilities of each of a set of models that define patterns of differential expression for each tuple. This approach begins by defining each of our models in terms of similarity and difference between samples. For a given model, we seek to define which samples behave similarly to each other, and for which sets of samples there are identifiable differences. In order to assess the posterior probabilities of each model for each tuple, we consider a distribution for the tuple defined by a set of underlying parameters for which some prior distribution exists. Samples behaving similarly to each other should possess the same prior distribution on the underlying parameters of the tuple, while samples behaving differently should possess different prior distributions. We develop our method based on the negative binomial distribution for the tuple data, and derive an empirical distribution on the set of underlying parameters from the whole of the data set.

An important advantage of our method is that the evaluation of posterior probability for multiple models is simply achieved. For this reason, the techniques described are developed in a very general form.

### Model definitions

In forming a set of models for the data, we consider which patterns are biologically likely. In the simplest case of a pairwise comparison, we have count data from some samples from both condition *A *and condition *B*. If we suppose that we have two biological replicates for each condition, then there are four libraries, *A*_1_, *A*_2_, *B*_1_, *B*_2_, where *A*_1_, *A*_2 _and *B*_1_, *B*_2 _are the replicates. In most cases, it is reasonable to suppose that at least some of the tuples may be unaffected by our experimental conditions *A *and *B*. The count data for each sample in these tuples will then share the same underlying parameters. However, some of the tuples may be influenced by the different experimental conditions *A *and *B*. For such a tuple, the data from samples *A*_1 _and *A*_2 _will share the same set of underlying parameters, the data from samples *B*_1 _and *B*_2 _will share the same set of underlying parameters, but, crucially, these sets of parameters will not be identical. We can thus treat our models as non-overlapping sets of samples. Our first model, of no differential expression, is thus defined by the set of samples {*A*_1_, *A*_2_, *B*_1_, *B*_2_}. Our second model, of differential expression between condition *A *and condition *B *is defined by the sets {*A*_1_, *A*_2_} and {*B*_1_, *B*_2_}.

#### More complex models

In the simple example described, only two models are plausible, and this framework may seem overly complex. However, in experimental designs involving multiple sample groups, many more models are possible. As an example, we consider the next most complex experimental design, involving samples from three distinct conditions *A*, *B *and *C*. In this case, for a given tuple, either the data are equivalently distributed across all samples, or they are equivalently distributed under two conditions but not under the third, or they are differently distributed in all three conditions. There are thus five models which we need to consider.

In the first of these, all samples are equivalently distributed, and so the model is defined by the set {*A*_1_, *A*_2_, ..., *B*_1_, *B*_2_, ..., *C*_1_, *C*_2_, ...}. We then need to consider the three models under which there is equivalent distribution under two conditions but not the third. The first of these models can be described by the sets {*A*_1_, *A*_2_, ..., *B*_1_, *B*_2_, ...}, {*C*_1_, *C*_2_, ...}, in which the data from condition *A *and condition *B *are distributed equivalently, and the data from condition C are differently distributed. Similarly, we need to consider the other two models in which a single condition differs from the other two, {*A*_1_, *A*_2_, ..., *C*_1_, *C*_2_, ...}, {*B*_1_, *B*_2_, ...} and {*B*_1_, *B*_2_, ..., *C*_1_, *C*_2_, ...}, {*A*_1_, *A*_2_, ...}. Finally, we need to consider the model defined by the sets {*A*_1_, *A*_2_, ... }, {*C*_1_, *C*_2_, ... }, {*A*_1_, *B*_2_, ...}, in which the data from all three conditions are differently distributed.

It is clear from considering even this relatively simple example that the number of potential models rises rapidly as the number of different experimental conditions increases. We should also note, however, that in many cases we will be able to exclude particular models based on biological knowledge (if, for example, we know that condition *B *is a subtype of condition *A*, we might exclude the model defined by {*A*_1_, *A*_2_, ..., *C*_1_, *C*_2_, ...}, {*B*_1_, *B*_2_, ...}), and so the complexity of the system need not grow too rapidly. Our task is now to determine the posterior probability of each of our models, given the data, for each tuple. This will allow us to form ranked lists of the tuples, ordered by the posterior probabilities of a particular model (for instance, a model of differential expression between experimental conditions).

One interesting advantage of determining posterior probabilities, rather than significance values (*p*-values) for each comparison, is that, since we acquire posterior probabilities for each model and each tuple, and since these models are mutually exclusive, it is trivial to combine models of interest by summing the posterior probabilities. For example, if we are interested not in any specific type of differential expression, but simply in whether or not differential expression of any type exists in our data, we can acquire the probability of differential expression of any type by summing the posterior probabilities of all (biologically plausible) models that describe differential expression. We can then rank the tuples on these probabilities as well as on the probabilities of individual models.

### Equivalence of distributions

Suppose we have the count data from a set of *n *samples A= {*A*_1_, ..., *A*_*n*_}, such that the observed data for a particular tuple, *c*, is given by (*u*_1*c*_, ..., *u*_*nc*_) where *u*_*ic *_is the count for a particular tuple *c *for sample *i*. For each sample *A*_*i*_, we also have the library size scaling factor *l*_*i*_. For each tuple, then, we can consider the data to be

$$D_{c}=\{(u_{lc},\cdots u_{nc}),(l_{1},\cdots ,l_{n})\}$$

Now we consider some model *M *on these data defined by the sets {*E*_1_, ..., *E*_*m*_}. If, in this model, the samples *A*_*i *_and *A*_*j *_are in the same set *E*_*q*_, then we know that they have the same parameters of underlying distribution *θ*_*q*_. We can define a set *K *= {*θ*_1_, ..., *θ*_*m*_}. For notational simplicity, we will also define the data associated with the set *E*_*q *_as *D*_*qc *_= {(*u*_*ic *_: *A*_*i *_∈ *E*_*q*_), (*l*_*i *_: *A*_*i *_∈ *E*_*q*_)} Given a model *M *for the data, then the quantity of interest for each tuple *c *is the posterior probability of the model *M *given the data *D*_*c*_, that is

(1) $$\mathbb{P}(M|D_{c})=\frac{\mathbb{P}(D_{c}|M)\mathbb{P}(M)}{\mathbb{P}(D_{c})}$$

We can then attempt to calculate ℙ(*D*_*c *_|*M*) by considering the marginal likelihood

(2) $$\mathbb{P}(D_{c}|M)=\int \mathbb{P}(D_{c}|K,M)\mathbb{P}(K|M)\text{d}K$$

### Negative binomially distributed data

There are a number of possible distributions which could be used for *D*_*c*_|*K*, *M *and *K*|*M*. One approach that seems natural is to assume that the data are Poisson distributed and the parameters Gamma distributed, thus modelling the rarity of any individual molecule being sequenced and allowing a form of the Poisson-Gamma conjugacy to be used in calculating ℙ(*D*_*c *_|*M*). However, as Robinson and Smyth [8] point out, this model fails to take into account the extra variability introduced by biological replication. An assumption that the data are negative binomially (over-dispersed Poisson) distributed may be used to account for this variability. Robinson and Smyth [9] showed the existance of over-dispersion in real data, and we are also able to see this in the data set we introduce below. Furthermore, Lu *et al *[7] show in simulated data that an assumption of a negative binomial distribution can be robust even if the data are not truly negative binomially distributed.

In the case of equal library sizes, it is possible under an assumption of a negative binomial distribution to develop an exact test for the likelihood of observing the data given non-differential expression. The problem of unequal library sizes can be approached by generating 'pseudodata' that is approximately identically distributed to the real data but has a common library size. This is the approach taken by Robinson and Smyth [9]. As an alternative to this approach, we use numerical methods in an empirical Bayesian approach that allows us to retain the real data, using library size as a scaling factor. We consider a sample *A*_*i *_belonging to the set *E*_*q *_with library size *l*_*i*_. We now assume that the count in this sample at tuple *c*, *u*_*ic *_is distributed negative binomially, with mean *μ*_*q*_l_*i *_and dispersion *ϕ*_*q*_, where *θ*_*q *_= (*μ*_*q*_, *ϕ*_*q*_). Then one parametrization can be defined as

$$\mathbb{P}(u_{ic};l_{i},\phi_{q},\mu_{q})=\frac{\Gamma (u_{ic}+\phi_{q}^{-1})}{\Gamma (\phi_{q}^{-1})u_{ic}!}{\left(\frac{1}{1+l_{i}\mu_{q}\phi_{q}}\right)}^{\phi_{q}^{-1}}{\left(\frac{l_{i}\mu_{q}}{\phi_{q}^{-1}+l_{i}\mu_{q}}\right)}^{u_{ic}}$$

There is unfortunately no obvious conjugacy that can be applied as in the Poisson-Gamma case. However, if we can define an empirical distribution on *K *then we can estimate ℙ(*D*_*c *_| *M*) numerically. We assume first that the *θ*_*q *_∈ *K *are independent with respect to *q*. Then

$$\begin{array}{l} \mathbb{P}(D_{c}|M)\;=\;\int \mathbb{P}(D_{c}|K,M)\mathbb{P}(K|M)\text{d}K\\ \;\;\;\;\;\;\;=\;\prod_{q}\int \mathbb{P}(D_{qc}|\theta_{q})\mathbb{P}(\theta_{q})\text{d}\theta_{q} \end{array}$$

This assumption reduces the dimensionality of the integral and thus improves the accuracy of the numerical approximation to the integral.

Next we suppose that for each *θ*_*q *_∈ *K *we have a set of values Θ_*q *_that are sampled from the distribution of *θ*_*q*_. Then we can derive the approximation [13]

(3) $$\mathbb{P}(D_{c}|M)\approx \prod_{q}\frac{1}{\left|\Theta_{q}\right|}\;\;\sum_{\Theta_{q}}\;\;\left[\prod_{\left\{i:A_{i}\in E_{q}\right\}}\frac{\Gamma (u_{ic}+\phi_{q}^{-1})}{\Gamma (\phi_{q}^{-1})u_{ic}!}{\left(\frac{1}{1+l_{i}\mu_{q}\phi_{q}}\right)}^{\phi_{q}^{-1}}{\left(\frac{l_{i}\mu_{q}}{\phi_{q}^{-1}+l_{i}\mu_{q}}\right)}^{u_{i}}\right]$$

The task that then remains is to derive the set Θ_*q *_from the data.

#### Empirically derived distributions on K

We can derive an empirical distribution on *K *by examining the whole dataset. For each set of samples *E*_*q*_, we would like to find some estimate of the mean and dispersion of the distribution underlying the data from a single tuple, *D*_*qc*_. By similarly finding estimates of the mean and dispersion for a large number of tuples, we would have our sampling Θ_*q*_. The chief difficulty here lies in properly estimating the dispersion. For example, suppose that the data from a given tuple shows genuine differential expression. If the model that we are testing assumes that there is no differential expression, then the dispersion will be substantially over-estimated for this tuple. Since we do not know in advance which tuples are genuinely differentially expressed and which are not, we need to consider the replicate structure of the data in order to properly estimate the dispersions. We define the replicate structure by considering the sets {*F*_1_, ... *F*_*s*_} where *i*, *j *∈ *F*_*r *_if and only if sample *A*_*j *_is a replicate of *A*_*i*_.

Given this structure for the data, we can estimate the dispersion of the data in a tuple *D*_*c *_by quasi-likelihood methods [14]. Quasi-likelihood methods have been shown to give good estimations of the dispersion of a single tuple in this setting [8]. We first define ${\overset{\wedge }{\mu }}_{rc}=\langle \left\{\frac{u_{ic}}{l_{i}}:i\in F_{r}\right\}\rangle$, and then choose *ϕ*_*c *_such that

(4) $$\begin{array}{ll} 2\;\sum_{r}\sum_{i\in F_{r}}\left\{u_{ic}\log\left[\frac{u_{ic}}{l_{i}{\overset{\wedge }{\mu }}_{rc}}\right]-\left(u_{ic}+\phi_{c}^{-1}\right)\log\left[\frac{u_{ic}+\phi_{c}^{-1}}{l_{i}{\overset{\wedge }{\mu }}_{rc}+\phi_{c}^{-1}}\right]\right\}\; & =\;\;\;n-1 \end{array}$$

Taking this value for *ϕ*_*c *_we can then re-estimate the values ${\hat{\mu }}_{ic}$ by maximum likelihood methods, choosing the values for ${\overset{\wedge }{\mu }}_{ic}$ that maximise the likelihoods

$$\mathbb{P}\left(\left\{u_{ic}:i\in F_{r}\right\};l_{i}:i\in F_{r},\phi_{c},{\overset{\wedge }{\mu }}_{rc}\right)=\prod_{i\in F_{r}}\;\;\frac{\Gamma \left(u_{ic}+\phi_{c}^{-1}\right)}{\Gamma \left(\phi_{c}^{-1}\right)u_{ic}!}{\left(\frac{1}{1+l_{i}{\overset{\wedge }{\mu }}_{rc}\phi_{c}}\right)}^{\phi_{c}^{-1}}{\left(\frac{l_{i}{\overset{\wedge }{\mu }}_{rc}}{\phi_{c}^{-1}+l_{i}{\overset{\wedge }{\mu }}_{rc}}\right)}^{u_{ic}}$$

for each *r*.

We then iterate on our estimations of *ϕ*_*c *_and ${\hat{\mu }}_{ic}$ until we achieve convergence.

This gives us a value for *ϕ*_*c*_. We then need to estimate the mean of the distribution underlying the data *D*_*qc*_, that is, for the set of samples in *E*_*q*_, which we can easily do by fixing the value acquired for *ϕ*_*c *_and estimating the mean *μ*_*qc *_by maximum likelihood methods, choosing the value for *μ*_*qc *_that maximises the likelihood

$$\mathbb{P}\left(D_{qc},\phi_{c},\mu_{qc}\right)=\prod_{\left\{i:A_{i}\in E_{q}\right\}}\frac{\Gamma \left(u_{ic}+\phi_{c}^{-1}\right)}{\Gamma \left(\phi_{c}^{-1}\right)u_{ic}!}\;\;{\left(\frac{1}{1+l_{i}\mu_{qc}\phi_{c}}\right)}^{\phi_{c}^{-1}}{\left(\frac{l_{i}\mu_{qc}}{\phi_{c}^{-1}+l_{i}\mu_{qc}}\right)}^{u_{ic}}$$

for each q.

We can then form the set Θ_*q *_= {(*μ*_*qc*_, *ϕ*_*c*_)} by repeating this process for multiple *h*, and are then able to calculate ℙ(*D*_*c *_| *M*) from Eqn 3.

This method of estimating the dispersion assumes that the dispersion of a tuple is constant across different sets of samples. In most cases, where the number of samples is low, this is likely to be the best approach. Where there is some expectation that the dispersion will be substantially different between sets of replicates, there may be advantages to estimating the dispersions individually for each of the different sets of samples in each model, while still considering the replicate structure within these sets. This is easily done by restricting the data (and corresponding replicate structure) to *D*_*qc *_when estimating the dispersion in Eqn 4. We found no substantial differences between these approaches in simulation studies (unpublished data) and so show only the results acquired when the dispersion of each tuple is assumed constant.

### Estimation of prior probabilities of each model

A number of options are available when considering the prior probabilities of each model ℙ(*M*) required in Eqn 1. If we are able to estimate these from other sources, this may provide the optimum solution. However, in many cases we may not be able to provide a reasonable estimate of prior probabilities. We propose that the methods suggested by Smyth [15] for estimating proportions of differentially expressed genes in analysis of microarray experiments may reasonably be adapted to estimate these priors. We begin by choosing (ideally based on our prior knowledge about the models) some value *p *to use as the prior probability for the model *M *in order to estimate the posterior probability ℙ(*M *| *D*_*c*_) for the *c*th tuple. But then we can derive a new estimate

$$p^{\prime }\;=\;{\langle \mathbb{P}(M|D_{c})\rangle }_{c}$$

for the prior probability of model *M*. By iterating until convergence, we acquire estimates of the prior probabilities for each model. In practice, we find that the initial choice of the *p*s has no substantial effect on the values to which they finally converge. This method is straightforward to implement, but potentially allows for positive feedback and hence over-estimation of the prior probability of a model (and corresponding under-estimation of the prior probabilities of the other models).

An alternative to this approach would be to establish some distribution on the prior probabilities of our models and find the marginal posterior probability of the data based on this distribution. One approach to this might be to use the distribution of posterior probabilities as an approximation to a distribution on the priors. We could then use a numerical integration method to re-estimate the posterior probabilities, and iterate as before. However, in practice this method is extremely computationally intensive and offers little improvement in the accuracy of the predictions made (unpublished data).

### The scaling factor ℙ(*D*_*c*_)

Finally, we need to consider the scaling factor ℙ(*D*_*c*_) in Eqn. 1. Since the number of possible models on *M *on A is finite, though potentially large, the scaling factor ℙ(*D*_*c*_) can be determined by summing over all possible *M*, given appropriate priors ℙ(*M*). In practice, the number of models may be limited by only considering those that are biologically plausible, or by imposing some distribution on the number of sets in *M *in a similar manner to Lönnstedt *et al*'s approach [16] for analysis of variance in microarray data.

## Results and Discussion

We use both simulated and real data to compare the method we have developed to the previously developed methods of Robinson and Smyth [9] as implemented in the edgeR [17,18] (version 1.4.7) Bioconductor [19] package, the overdispersed log-linear model of Lu *et al *[7], the overdispersed logistic model of Baggerly *et al *[6], and the recently released methods DEGseq[10] (version 1.2.2) and DESeq[11] (version 1.0.4). We compare these methods to our empirical Bayes approach as implemented in the R package baySeq (version 1.1.23), with the default settings used for the baySeq and edgeR packages. Overall, we found that the default settings of the edgeR package seem to give good performance. Alterations to the default settings, in particular to the 'moderation' parameter, caused some small improvements in performance for some simulations but degraded it slightly in others. We have, therefore, used the default settings here as in real-world applications it will be difficult to determine how to alter these settings to optimise performance. The recommended method of operation for the DESeq package is to infer library sizes from the data. However, we observed that this gave extremely poor performance in simulations in which a large proportion of the data are differentially expressed in a single direction. We therefore use the known library sizes in the implementation of the DESeq method, as we also do for all other methods, with the exception of DEGseq, which does not accept library size as a parameter. The DEGseq package has multiple modes of operation; we found that the MA plot-based method with random sampling (MARS) performed best on simulated data (unpublished data) and have therefore used this approach (with default settings otherwise) in the comparison studies.

### Comparison of methods for pairwise comparisons: simulated data

We begin by applying the methods being evaluated to the simulation studies described in Robinson and Smyth [9]. We choose to replicate these simulation studies, and the manner in which the results are presented, in order to allow direct comparisons between our method and previous approaches to this problem. The purpose of these simulations is to establish the ability of the methods to rank the tuples in order of differential expression and evaluate the number of true and false positives for the top *N *tuples.

#### Random dispersion simulations

Robinson and Smyth [9] suggest one possible simulation for high-throughput sequencing count data. The library sizes, *l*_*i*_, are sampled from a uniform distribution between 30000 and 90000. These library sizes are considerably smaller than those available from the current generation of sequencing technologies. However, increasing the library size to better reflect current levels does not significantly alter the conclusions drawn, because the 'library size' is, in effect, a scaling factor. All tuples are simulated from a negative binomial distribution, and we simulate differential expression by varying the means of the distribution from which they are sampled.

For a non-differentially expressed tuple *c*, we simulate the data with means *λ*_*c*_*l*_*i *_where the *λ*_c _are sampled randomly from a a set of values empirically estimated by the edgeR method from a SAGE dataset consisting of both normal and cancerous cells [20].

Ten percent of the ten thousand simulated tuples are differentially expressed. In order to produce both over and under-expression in our simulated data, we simulate the differentially expressed data in one of two ways, where the alternatives are chosen at random for each tuple. We can simulate the data for the first *n*_1 _samples with means $\lambda_{c}l_{i}/\sqrt{b}$ while the data from the remaining *n*_2 _samples are simulated with mean $\lambda_{c}l_{i}\sqrt{b}$ Alternatively, we can simulate the data for the first *n*_1 _samples with mean $\lambda_{c}l_{i}\sqrt{b}$ while the data from the remaining *n*_2 _samples are simulated with mean $\lambda_{c}l_{i}/\sqrt{b}$.

Small (*n*_1 _= *n*_2 _= 2) and moderate (*n*_1 _= *n*_2 _= 5) numbers of libraries are compared, with large (*b *= 8) and moderate (*b *= 4) differential expression. Dispersions are randomly sampled from a gamma distribution with shape = 0.85 and scale = 0.5.

**Figure 1:**
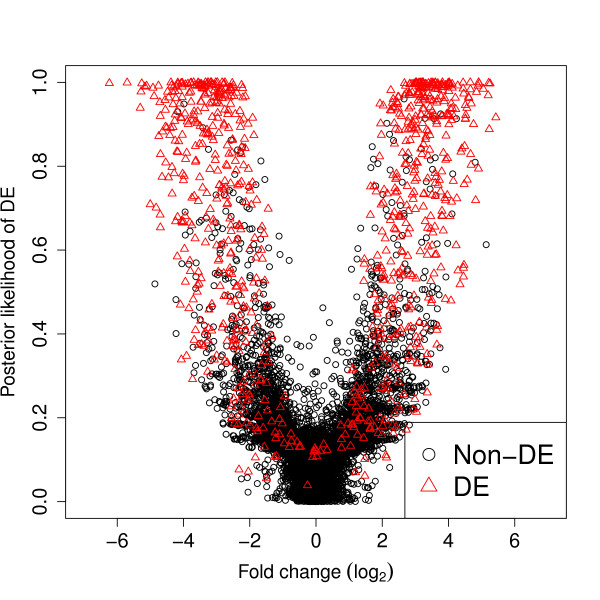
**Estimated posterior probabilities of differential expression against observed fold-change**. Estimated posterior probabilities of differential expression against observed fold-change from a single simulation of ten thousand tuples, of which one thousand are truly differentially expressed (DE) and nine thousand are not differentially expressed (non-DE).

For the baySeq method, posterior probabilities were calculated for each tuple for each of two models, one defining differential expression between the first *n*_1 _libraries and the second *n*_2 _libraries and one defining no differential expression between any library. Figure 1 shows the estimated posterior probability of differential expression plotted against the estimated log fold change for a single simulation with *b *= 8 and *n*_1 _= *n*_2 _= 5. We see a 'wine glass' shaped plot, characteristic of this analysis.

The 'stem' of the goblet is made up of tuples with low fold change and reasonably high levels of expression. With these tuples, it is relatively easy to identify them as non-differentially expressed, and so these tuples have low posterior probability of differential expression. However, some tuples with low fold change also have very low absolute values. With low absolute values in a tuple, it becomes harder to determine whether or not the tuple is genuinely differentially expressed or not, and so these values tend to have slightly higher posterior probabilities of differential expression than tuples with high absolute values but low fold change. The top of the stem, with a posterior probability of differential expression of around 0.2, is thus composed of tuples that have only one or two counts observed in any sample. For these very low expression tuples, changes of only one or two counts in a sample can lead to a relatively large fold change difference. However, these small changes do not substantially affect the posterior probability and so, although we see a spread in the fold change at the top of the stem, the posterior probability of differential expression remains low for these tuples. We tend not to see a similar spread for the tuples near the base of the stem as these tuples tend to have a high expression. For a tuple with a high expression to show a high fold change, but nevertheless have a low posterior probability of differential expression, there must be a very high dispersion associated with such a tuple, which will not often occur.

In the arms of the wine glass, we see that as the fold change increases, the posterior probability of differential expression also increases, although there is a wide range of posterior probabilities for (for example) a fold change of 4. We see this range of posterior probabilities of differential expression for a given fold change as the posterior probability also depends heavily on both the dispersion observed within the data, and the level of expression of the tuple, since, as before, it is easier to tell whether or not a highly expressed tuple is genuinely differentially expressed or not. For high posterior probabilities of differential expression, we see an increased density of tuples, predominately consisting of truly differentially expressed tuples.

**Figure 2:**
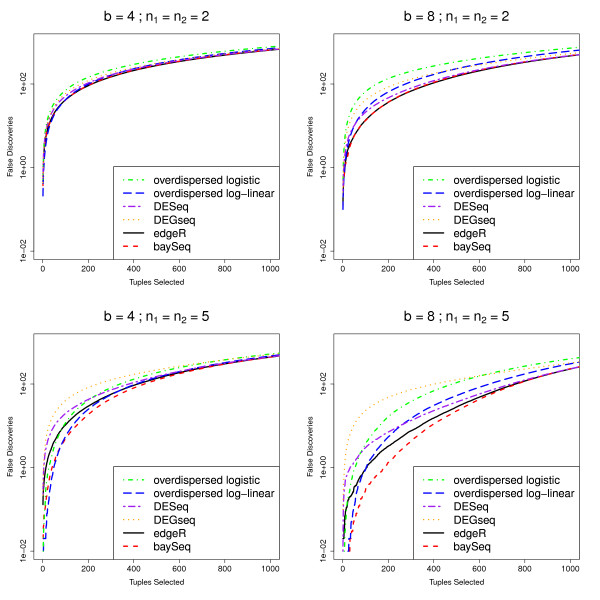
**Mean FDR curves for different numbers of libraries and degrees of differential expression**. Mean FDR curves, based on 100 simulations, comparing the performance of multiple methods in identifying pairwise differential expression. The data contain 1000 truly DE tuples and 9000 non-DE tuples and are simulated with varying number of libraries *n*_1 _and *n*_2_, different degrees of differential expression *b*, and randomly chosen dispersions for each tuple (~ Γ (0.85, 0.5)).

As in Robinson and Smyth [9], false discovery rate (FDR) curves are used to assess the ability of the methods to successfully rank the tuples. False discovery rates for these data are calculated by [9] on the basis of one simulation. For increased robustness, we estimate mean false discovery rates for the top *N *tuples over 100 simulations (Figure 2). For the baySeq method, the tuples were ordered by the posterior probability of differential expression and true and false positive rates were calculated on the basis of this ordering. For the edgeR, the overdispersed log-linear, overdispersed logistic, DESeq and DEGseq methods, the tuples were ordered on the basis of the *p*-values estimated by each method.

In these simulations, the baySeq method appears to perform as well or better than the existing methods. The performance of the baySeq approach is virtually identical to that of edgeR for small numbers of libraries (*n*_1 _= *n*_2 _= 2). For larger numbers of libraries, baySeq appears to offer an improvement in performance over edgeR. For small *b*, the overdispersed log-linear approach seems to show comparable performance to edgeR and baySeq. For larger *b*, however, particularly for higher numbers of selected tuples, the edgeR and baySeq methods perform considerably better than the log-linear approach. The log logistic, DESeq and DEGseq methods always perform poorly compared with both the edgeR method and the baySeq approach.

To establish whether this difference in performance for these methods is meaningful in a practical sense, we estimate from these analyses that if we were to validate the top 200 tuples identified by edgeR, baySeq, and the overdispersed log-linear model fit, for *n*_1 _= *n*_2 _= 2, *b *= 4 we would expect 92.66 false positives for the baySeq method, 91.13 from edgeR and 98.65 for the overdispersed log-linear approach. For *n*_1 _= *n*_2 _= 2, *b *= 8, we would expect 36.88, 36.46, and 64.43 false positives from baySeq, edgeR and the overdispersed log-linear approaches respectively. However, for the higher numbers of libraries, where *n*_1 _= *n*_2 _= 5, for *b *= 4 we expect 18.60, 29.44 and 24.74 false positives, while for *b *= 8 we expect 1.33, 3.25 and 5.42 false positives from the baySeq method, edgeR and the overdispersed log-linear approach respectively. For higher numbers of libraries, therefore, we achieve a practically meaningful improvement by using the baySeq method.

#### Fixed dispersion simulations

For completeness of comparison with previous methods, we also consider a less realistic simulation first developed by Lu *et al *[7]. We simulate ten library sizes as before. The tuples are again simulated from a negative binomial distribution but now with a fixed dispersion *ϕ *of either 0.17, 0.42 or 0.95. 5000 non-differentially expressed tuples are simulated with mean *λl*_*i*_, and 5000 tuples are chosen to be differentially expressed; those from libraries 1-5 are again simulated with mean *λl*_*i *_while those from libraries 6-10 are simulated with mean *bλl*_*i*_, and so we see only over-expression of libraries 6-10 in the data. These simulations are applied with *λ *= 0.0002 and *b *= 4.

**Figure 3:**
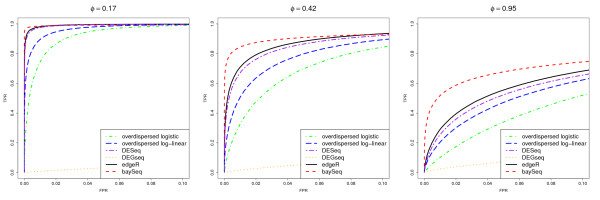
**Mean ROC curves for data with constant dispersion**. Mean ROC curves, based on 100 simulations, comparing the performance of multiple methods in identifying pairwise differential expression. The data contain 5000 truly DE tuples and 5000 non-DE tuples and are simulated from a negative binomial distribution with constant dispersion for all tuples *ϕ *= 0.17, 0.42 or 0.95.

As in Robinson and Smyth [9], we examine the results by considering receiver-operating characteristic (ROC) curves for all analyses (Figure 3). The performance of the DEGseq methods is strikingly poor. Further investigation showed that this loss of performance is associated with the large proportion of tuples that are differentially expressed in the same direction, that is, all up-regulated in libraries 6-10. If either the proportion of differentially expressed tuples is reduced sufficiently, or if similar proportions of up-regulation and down-regulation exist in the data, then the performance the DEGseq method improves substantially. This poor performance occurs becuase of the assumption by the DEGseq method that the mean of the log-ratios between samples is approximately zero. In this case, because the differential expression always occurs in the same direction, this assumption fails. This may be a problem in real-life applications if large numbers of genomic features are all affected similarly.

Of the remaining methods, we see that as the dispersion increases, the performance of all the methods decreases; however, the baySeq approach appears to outperform all existing methods for all values of *ϕ*, in that, for low false positive rates, the baySeq method has higher true positive rates. This effect is particularly noticeable for simulations involving higher dispersion. The overdispersed logistic model in general performs worse than the overdispersed log-linear method. In turn, the overdispersed log-linear approach is outperformed by the DESeq method, which is outperformed by the edgeR method. This roughly corresponds to the relative performance of these methods on the more realistic simulations.

### Comparison of methods for pairwise comparisons: biological data

We next apply the methods to a set of data acquired by Illumina sequencing small RNAs (20-24 nucleotide) from leaf samples of *Arabidopsis thaliana *(Gene Expression Omnibus accession number GSE16959). The experimental data are taken from two wild-type samples and two RDR6 (RNA-dependent RNA polymerase 6) knockout samples. It is known that RDR6 is required for production of tasRNAs (trans-acting small RNAs) [21]. We would therefore expect to see differential expression of tasRNAs in a comparison between the wild-type and the mutant samples; specifically, under-expression of tasRNA associated small RNA sequences in the RDR6 knockouts.

**Figure 4:**
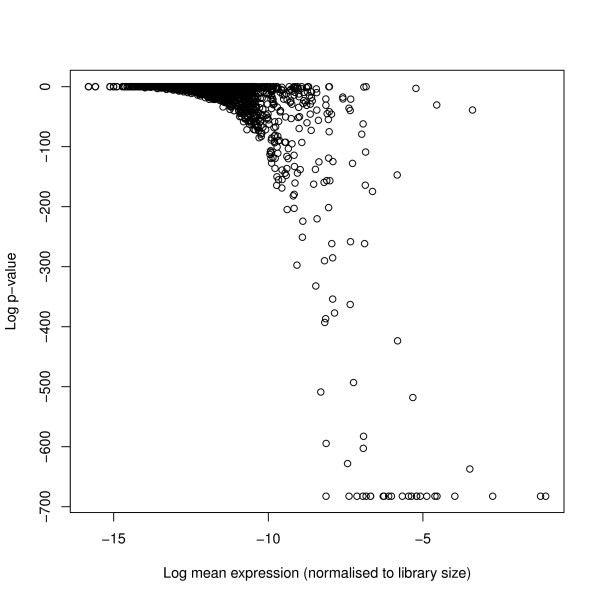
**(Log) p-values of real sequence data under null hypothesis of no overdispersion against mean expression levels of each sequence**. (Log) p-values of real sequence data under the null hypothesis of no overdispersion and alternative hypothesis of overdispersion. We acquire these for each sequence by performing likelihood-ratio tests on the fit of a Poisson model and an alternative negative binomial model, allowing for both differences in library size and between the two sample types. Although a number of sequences show no significant variation from the Poisson model, a substantial number show very significant variation. The sequences for which overdispersion is particularly significant are those with high mean expression levels, as these are the sequences for which overdispersion can most easily be detected.

We consider only those sequence reads that perfectly matched the *Arabidopsis *genome as defined by The Arabidopsis Information Resource (TAIR) [22] (version 9). Sequences were matched using the PatMaN algorithm [23]. A total of 70619 unique small RNA sequences matching the genome were observed in the data, and the total number of genome matching reads, used to define the library sizes, were 1840563, 594356, 1477155 and 276006 for the two wildtype and two RDR6 mutant knockout samples respectively. We examined this data for overdispersion by performing likelihood-ratio tests on the reads acquired for each sequence by fitting both a Poisson model and an alternative negative binomial model, allowing for both differences in library size and between the two sample types. Although many sequences showed no significant variation from the Poisson model, a substantial number showed very significant variation (Figure 4). This effect is noticeable particularly in those sequences which have a high average count, presumably because it is for these sequences that overdispersion can reasonably be detected.

We identified 678 different small RNA sequences that perfectly matched the tasRNA loci (TAS1a, TAS1b, TAS1c, TAS2, TAS3 and TAS3b) and matched nowhere else in the genome. 21 of these small RNA sequences showed higher expression in the RDR6 mutant than in the wild-type samples and these were excluded, leaving 657 potential true positives. We applied the methods to the count data for each small RNA sequence, seeking differential expression between the wild-type samples and the RDR6 knockout samples. We then ranked the sequences by the extent to which they are reported as differentially expressed by each method. We would expect a sizeable fraction of our 657 potential true positives to appear near the top of the list.

**Figure 5:**
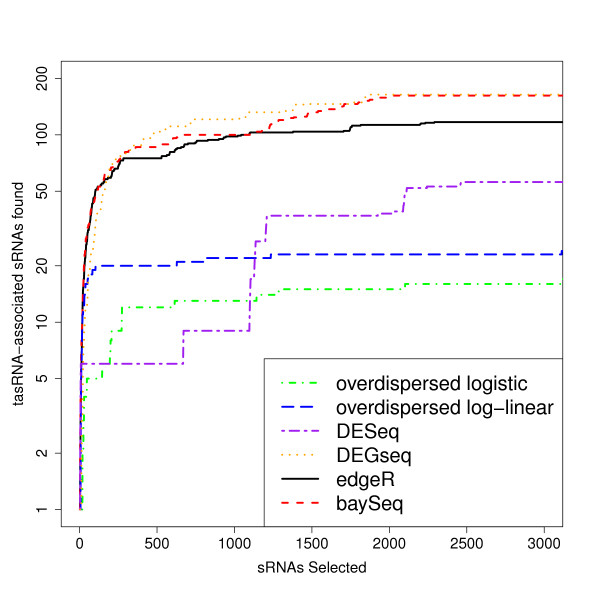
**Number of tasRNA-associated small RNAs identified as differentially expressed in RDR6 knockout experiment**. Number of tasRNA-associated small RNAs against the number of differentially expressed small RNAs at the top of each list acquired by each method in an analysis of small RNA data from two wild-type samples and two RDR6 knockout samples. We expect tasRNA-associated small RNAs to be under-expressed in the RDR6 knockout samples, and hence to find these amongst the differentially expressed tuples.

Figure 5 shows the number of tasRNA associated sequences that are identified by the various methods against the number of small RNA sequences selected as differentially expressed for the top three thousand small RNA sequences. Both edgeR and baySeq identify considerably more tasRNA-associated small RNAs than the DESeq method and the overdispersed logistic and overdispersed log-linear approaches, with the overdispersed logistic model performing particularly poorly. The baySeq method in general identifies more tasRNA associated small RNA sequences than edgeR for a given number of selected small RNA sequences. Perhaps surprisingly, DEGseq does well in this comparison, identifying only slightly fewer tasRNA-associated small RNAs than baySeq and edgeR for low numbers of selected small RNAs, and slightly more tasRNA-associated small RNAs once the number of small RNAs selected is greater than 500.

### Multi-group experimental designs

We next illustrate the application of our method to a more complex experimental design involving multiple experimental conditions. We return to the example discussed in the Methods section, in which we have sequence data from three conditions; condition *A*, condition *B *and condition *C*, with *n *libraries from each condition. There are five different models for these data; one in which there is no differential expression of any kind, three models in which one of the conditions shows differential expression compared to the other two conditions, and one model in which data from all three conditions are different from each other.

We investigate the ability of our method to detect such patterns of differential expression by adapting the more realistic simulations proposed by Robinson and Smyth [9]. In total, data from 3*n *libraries are simulated, of which two thousand tuples are in some manner differentially expressed. The library sizes, and dispersions of each tuple are simulated as before, as are tuples with no true differential expression.

Five hundred tuples are simulated to have equivalently distributed data between condition *A *and condition *B*, with data from condition *C *differently distributed. In order to simulate both over and under-expression in the data, we simulate the data in one of two ways, where the alternatives are chosen at random for each tuple. We can simulate the data from condition *A *and condition *B *from a distribution with mean $\lambda_{c}l_{i}/\sqrt{b}$ and the data from condition *C *from a distribution with mean $\lambda_{c}l_{i}\sqrt{b}$. Alternatively, we simulate the data from condition *A *and condition *B *from a distribution with mean $\lambda_{c}l_{i}\sqrt{b}$ and the data from condition *C *from a distribution with mean $\lambda_{c}l_{i}/\sqrt{b}$.

Another five hundred tuples are simulated similarly such that tuples have equivalently distributed data in conditions *A *and *C*, but differently distributed data in condition *B*, while a third five hundred tuples are simulated such that tuples have equivalently distributed data in conditions B and C, but differently distributed data in condition *A*.

A further five hundred tuples are simulated in such a way that the data from all three conditions are differently distributed. For a given tuple, we simulate data from condition *X*_1 _from a distribution with mean *λ*_*cli*_. For condition *X*_2_, we simulate from a distribution with mean $\lambda_{c}l_{i}2\sqrt{b}$, and for condition *X*_3 _we simulate from a distribution with mean $\lambda_{c}l_{i}2\sqrt{b}$ Conditions *A*, *B *and *C *are randomly allocated to be conditions *X*_1,_*X*_2_, *X*_3 _for each tuple, and so we see various patterns of differential expression between these samples.

We again evaluate the methods by looking at the false discovery rates. In this analysis, we are interested in the ability of our method to accurately identify each of the different types of differential expression by simultaneously considering all possible models for the data. We can also consider the ability of our method to detect differential expression of any kind by taking the sum, for each tuple, of the posterior probabilities of all five models describing differential expression. We can thus consider four FDR curves for each type of differential expression present in the data, and an additional FDR curve for data showing differential expression of any kind.

For the pre-existing methods, in the overdispersed log-linear and the overdispersed logistic approaches, we are able to form linear models that describe all possible patterns of differential expression present in the data. For the edgeR, DEGseq and DESeq methods, we are only able to carry out pairwise comparisons and so we carry out three analyses on each dataset, one for each pattern of differential expression in which a single experimental condition is differentially expressed when compared to the other two. We are unable to consider directly, by the method of pairwise comparisons, the pattern of differential expression in which all three experimental conditions are differentially expressed, and so we do not use the edgeR, DEGseq or DESeq methods for the identification of tuples of this type.

**Figure 6:**
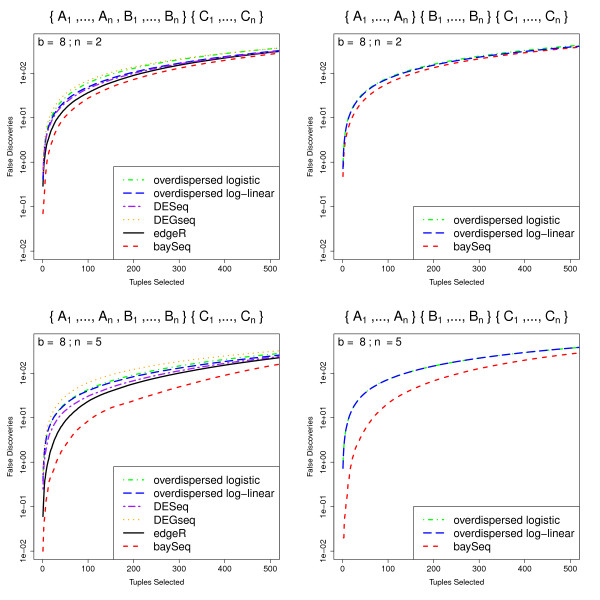
**Mean FDR curves for analyses of more complex experimental designs**. Mean FDR curves, based on 100 simulations, comparing the performance of multiple methods in identifying more complex patterns of differential expression. The data are simulated from samples coming from three experimental conditions *A*, *B *and *C*, giving a total of five possible patterns of differential expression. We show here the false discovery rates for the identification of tuples where one experimental condition differs from the other two ({*A*_1_, ..., *A*_*n*_, *B*_1_, ..., *B*_*n*_} {*C*_1_, ... *C*_*n*_}) and for the identification of tuples where all three experimental conditions are different ({*A*_1_, ..., *A*_*n*_}{*B*_1_, ... *B*_*n*_}{*C*_1_, ... *C*_*n*_}). The data are simulated with varying number of libraries *n *in each experimental condition.

We present the data (Figure 6) for *b *= 8 and *n *= 2 or *n *= 5. Again, for increased robustness, we estimate mean false discovery rates for the top *N *tuples over 100 simulations for all models. As would be expected, for all methods the false discovery rates are almost identical for the three models in which a single experimental condition is differentially expressed when compared to the other two conditions. We therefore show only the results for differential expression of conditions *A *and *B *compared with condition *C*, together with the results for the case where all three experimental conditions are differentially expressed. In this more complex experimental design, baySeq outperforms all existing methods, particularly as the number of libraries available increases. Perhaps surprisingly, the edgeR method does better than either the overdispersed log-linear or overdispersed logistic method in discovery of differential expression that can be expressed in terms of a pairwise comparison, as, to a lesser extent, does the DESeq method. The DEGseq method, however, does not perform as well as any of the alternatives in these comparisons.

**Figure 7:**
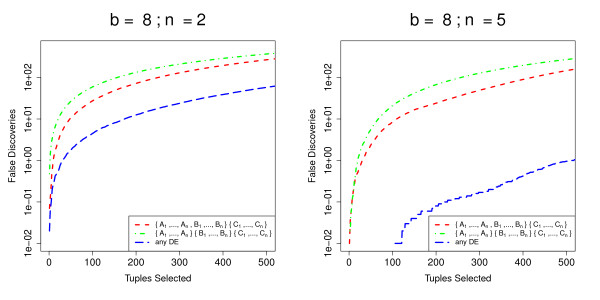
**Comparison of **baySeq**method's performance for different models in complex experimental designs**. Mean FDR curves, based on 100 simulations, comparing the performance of the baySeq method in identifying differential expression of different types in an analysis of more complex experimental designs. The data are simulated from samples coming from three experimental conditions *A*, *B *and *C*, giving a total of five possible patterns of differential expression. We show here the false discovery rates for the identification of tuples where one experimental condition differs from the other two ({*A*_1_, ..., *A*_*n*_, *B*_1_, ... *B*_*n*_}{*C*_1_, ... *C*_*n*_}) and for the identification of tuples where all three experimental conditions are different ({*A*_1_, ..., *A*_*n*_}{*B*_1_, ... *B*_*n*_}{*C*_1_, ... *C*_*n*_}). We also show false discovery rates for the identification of tuples showing differential expression of any kind.

Figure 7 shows how baySeq performs for the different models. The false discovery rate for the model in which all three experimental conditions differ from each other is considerably higher than that for pairwise comparisons, indicating the additional difficulty of fitting this more complex model. If we consider the suggestion described in the Methods section, of finding differential expression of any type by summing the posterior probabilities of all models describing differential expression, we see that the false discovery rate for tuples identified in this way is very low, particularly as the number of libraries available increases. This might suggest that some of the false discovery of the individual models may be due to differential expression of one type on occasion being mistaken for differential expression of another type.

## Conclusions

We present an empirical Bayes method, baySeq, that can simultaneously establish posterior probabilities of multiple models of differential expression and performs as well as or better than any existing techniques for identifying pairwise differential expression in count data. More significantly, this method enables the analysis of experimental designs involving multiple sample groups while using the whole data set to establish parameters on the level of dispersion present. This allows considerably greater accuracy in the analysis of more complex experimental designs than has previously been possible, and is hence a significant step forward in the analysis of the data being produced by high-throughput sequencing technologies. That the method produces posterior probabilities of models of differential expression, rather than significance values, offers a number of advantages in downstream analysis; for example, it becomes a simple matter to find an expected number of differentially expressed tuples, or to combine posterior probabilities of multiple models.

In developing this method, we have established a well-defined framework for describing diverse patterns of differential expression between samples. We then take an empirical Bayes approach in order to establish posterior probabilities of each model for each tuple. We achieve this by assuming that the data for each tuple is negative binomially distributed. This assumption is supported by the presence of over-dispersion in true data (Figure 4) and the work by Lu *et al *[7] showing that an assumption of a negative binomial distribution can be robust even if the data are not truly negative binomially distributed. We then estimate empirical prior distributions for the parameters of these negative binomial distributions. This is a very natural approach as high-throughput sequencing provides a large set of data from which to estimate prior distributions. An interesting feature of this approach is the flexibility we gain in choosing how to estimate the parameters of the negative binomial distributions. We have chosen to use quasi-likelihood methods here as they seem to give better performance than maximum-likelihood approaches (unpublished data). However, other methods of estimating these parameters (for example, Robinson and Smyth's [9] moderated conditional maximum likelihood, or Anders and Huber's [11] method for linking the variance of the negative binomial distribution to the mean) might be adapted to further improve the performance of our method. We can also deal easily with the problem of different library sizes, as this parameter can be built directly into the assumptions about the distribution of the data.

Our method is relatively computationally intensive, but has been implemented to take advantage of parallel processing, such that an analysis of pairwise differential expression of ten thousand tuples coming from ten samples takes approximately 7.5 minutes running on a machine with eight 2 GHz processors. We compare baySeq to the method implemented in the edgeR package, because this has been reported to outperform other existing approaches for pairwise comparisons [9], and is the most commonly used method for analysis of count data (based on Bioconductor download statistics). We also include comparisons to two recently developed methods for pairwise comparisons, DESeq and DEGseq, and to the older overdispersed logistic and overdispersed log-linear methods as these latter approaches allow for analysis of more complex experimental designs.

Comparisons of the methods on pairwise data are made on the basis of previously developed simulation studies [9], as well as on real biological data, and the baySeq method developed here performs comparably to, and in some cases better than any existing approach. We also see that one of the recently developed methods, DEGseq, shows extremely poor performance when there is a high proportion of unidirectional differential expression, although it is comparable to both edgeR and baySeq in other circumstances. When the dispersion of data is constant, the proportion of differentially expressed tuples is high, and the differential expression is unidirectional, there appears to be a clear improvement in performance by baySeq compared to all other methods using their default parameters (Figure 3).

For analyses of data with random dispersions (Figure 2), baySeq performs almost identically to edgeR for small numbers of libraries, but show a marked improvement in performance for larger numbers of libraries. The overdispersed log-linear method performs almost identically to baySeq for low levels of differential expression, but shows substantially worse performance for higher levels of differential expression. The DESeq and DEGseq methods show noticably worse performance compared to baySeq as both the level of differential expression and the number of libraries increases, with DEGseq performing particularly poorly. The overdispersed logistic method is always amongst the worst performers.

Analysis of real biological data again suggests that our method performs at least as well, and potentially better, than edgeR, while both methods appear to substantially outperform the overdispersed log-linear and logistic methods. The DESeq method again appears to perform poorly compared to baySeq. However, in these data DEGseq shows performance comparable to baySeq.

The chief advantage of the empirical Bayes method developed here, however, is its ready applicability to more complex experimental designs, although at present these methods remain limited to comparisons involving multiple groups, and are not able to account for, for example, paired samples. One possible extension to this work is thus the generalisation of the methods to some form of generalised linear model approach. However, our method is able to simultaneously identify multiple types of differential expression from a single experiment. In comparisons of the methods using simulations of an experimental design involving multiple groups (Figure 6), the baySeq method appears to offer substantial improvements over existing methods. Figure 7, which compares the performance of the baySeq method in identifying different patterns of differential expression, suggests that we should expect some loss of performance for the baySeq method for more complex patterns of differential expression. However, we can also see that combining models to acquire, for example, posterior probabilities of differential expression of any kind, is a valuable approach.

Our method thus provides performance as good as or better than previous methods whilst enabling experimenters to simultaneously consider many diverse sample types in a single sequencing experiment. We believe that this is a valuable approach representing an important step forward for the analysis of count data from sequencing experiments.

## Availability and Requirements

The empirical Bayes method developed in this paper are implemented in the software package baySeq[24] for the cross-platform computing environment R [25] (version 2.3 or greater). baySeq is released under the GPL-3 licence as part of the Bioconductor project [19] at http://www.bioconductor.org/packages/2.6/bioc/html/baySeq.html

## Authors' contributions

TJH designed and implemented the baySeq package and drafted the manuscript. KAK drafted the manuscript. All authors read and approved the final manuscript.
